# Reset-First and Multibit-Level Resistive-Switching Behavior of Lanthanum Nickel Oxide (LaNiO_3−x_) Thin Films

**DOI:** 10.3390/ma16144992

**Published:** 2023-07-14

**Authors:** Daewoo Kim, Jeongwoo Lee, Jaeyeon Kim, Hyunchul Sohn

**Affiliations:** Department of Materials Science and Engineering, Yonsei University, Seoul 03722, Republic of Korea; daewoo.kim@yonsei.ac.kr (D.K.);

**Keywords:** resistive random-access memory, rare-earth nickelates, LaNiO_3_, reset-first resistive switching, synaptic device, potentiation, depression

## Abstract

The resistive random-access memory (RRAM) with multi-level storage capability has been considered one of the most promising emerging devices to mimic synaptic behavior and accelerate analog computations. In this study, we investigated the reset-first bipolar resistive switching (RS) and multi-level characteristics of a LaNiO_3−x_ thin film deposited using a reactive magnetron co-sputtering method. Polycrystalline phases of LaNiO_3_ (LNO), without La_2_O_3_ and NiO phases, were observed at similar fractions of Ni and La at a constant partial pressure of oxygen. The relative chemical proportions of Ni^3+^ and Ni^2+^ ions in LaNiO_3−x_ indicated that it was an oxygen-deficient LaNiO_3−x_ thin film, exhibiting RS behavior, compared to LNO without Ni^2+^ ions. The TiN/LaNiO_3−x_/Pt devices exhibited gradual resistance changes under various DC/AC voltage sweeps and consecutive pulse modes. The nonlinearity values of the conductance, measured via constant-pulse programming, were 0.15 for potentiation and 0.35 for depression, indicating the potential of the as-fabricated devices as analog computing devices. The LaNiO_3−x_-based device could reach multi-level states without an electroforming step and is a promising candidate for state-of-the-art RS memory and synaptic devices for neuromorphic computing.

## 1. Introduction

Perovskite-type rare-earth nickelates, RNiO_3_ (R = La, Pr, Nd, Sm, …, Lu) [1,2], have attracted considerable attention because of their unique physical properties, such as metal–insulator transition capabilities [3] owing to their strong correlation of electrons. The energy-band structure of RNiO_3_ has a charge-transfer gap between the Ni 3d conduction band and the occupied O 2p valence band. The gap decreases gradually as the radius of R increases, owing to various driving forces, such as temperature and pressure, leading to charge transfer or a Mott transition [4,5].

Among RNiO_3_, LaNiO_3_ (LNO) is a highly conductive oxide with a perovskite-type structure, exhibiting an electrical resistivity of only a few hundred μΩ⋅cm to mΩ⋅cm at room temperature [6,7,8,9,10]. Therefore, LNO, owing to its crystallinity and high conductivity, has been primarily used as the bottom electrode (BE) in multilayer film devices, along with other ferroelectric, ferromagnetic, or multiferroic functional oxides [11,12,13]. However, achieving the actual stoichiometric composition of LNO films is challenging because Ni exists as Ni^3+^ and Ni^2+^ in the material. Additionally, the oxidation of Ni^2+^ to Ni^3+^ is accompanied by the creation of quasi-conductive oxygen vacancies owing to the overall charge neutrality requirement. The electrical conductivity and crystal structure of LaNiO_3−x_ (x ≥ 0) change with x [14,15,16]. In other words, the Ni^3+^/Ni^2+^ ratio of LNO affects its electrical transport properties. LaNiO_2.75_ with a Ni^3+^/Ni^2+^ ratio of one demonstrated semiconductive properties, whereas LaNiO_2.5_ with only Ni^2+^ showed insulating properties. Therefore, the conductivity of LaNiO_3−x_ decreases with increasing oxygen deficiency, and LaNiO_3−x_ acts as an insulator when x is 0.50.

Metal-oxide-based resistive random-access memories (RRAMs) [17,18] are promising nonvolatile memories [19,20,21]. Various binary oxides, such as Al_2_O_3_ [22,23], HfO_2_ [24,25,26], ZrO_2_ [27,28,29], Ta_2_O_5_ [30,31], and ZnO [32,33], have been studied for resistive-switching (RS)-memory devices owing to their relatively simple structure and ease of fabrication. Additionally, multicomponent perovskite oxides, such as Pr_0.3_Ca_0.7_MnO_3_ [34,35,36], have been widely examined for their nonvolatile, forming-free memory switching; area scalability; low variability; and good reliability. The RS behaviors of thin films formed by coating and pulsed laser deposition (PLD), which are different from conventional deposition methods such as physical vapor deposition (PVD) and atomic layer deposition (ALD), have also been reported [37,38,39]. Meanwhile, RRAM is promising as a synaptic device for neuromorphic computing, which has emerged as a solution to overcome the von Neumann bottleneck. RRAM-based synaptic devices can exhibit multi-level RS characteristics to mimic biological brain stimulation systems, and the simple stacked cross-point array structure enables tremendous scalability of memory devices. Various metal-oxide- and electrochemical-metallization (ECM)-based materials have been studied as RS materials with multibit-level properties [18,40,41]. However, studies on oxygen-deficient LaNiO_3−x_ thin films have been mainly focused on their physical properties, chemical composition, and conductivity, whereas RRAM devices with RS behavior and the ability to mimic biological synapse functions have not been reported. Therefore, this study investigated the RS characteristics of LaNiO_3−x_ thin films deposited by radio-frequency (RF)-magnetron co-sputtering using La and Ni targets. The reset-first RS behavior, achieved without an electroforming step, and the feasibility of constructing a multi-level device were demonstrated using a direct-current (DC) bias sweep. Additionally, the conductance gradually changed under consecutive AC pulse responses. The forming-free and multi-level RS behaviors of this ternary-material-based device render it promising as a synaptic device for neuromorphic computing applications [42,43,44].

## 2. Materials and Methods

TiN/LaNiO_3−x_/Pt stacks were fabricated as RS metal–insulator–metal (MIM) devices. First, Ti/TiN adhesive films with thicknesses of 10/50 nm were deposited onto SiO_2_/Si substrates using DC magnetron sputtering [45]. After depositing a Pt layer with a thickness of 100 nm as the BE, SiO_2_ was deposited to form a via-type pattern with an area of 2 × 2 μm^2^. Subsequently, LaNiO_3−x_ films with a thickness of approximately 15 nm were deposited on the BE using reactive RF-magnetron co-sputtering [46,47] with La and Ni targets. The base and working pressures of the main chamber during sputtering were less than 4 × 10^−4^ and 0.4 Pa, respectively. The oxygen partial pressure fraction was 25%, and the substrate temperature was maintained at 400 °C during deposition. Finally, a 100 nm thick TiN top electrode was fabricated using DC magnetron sputtering and a lift-off process. The crystallinity of LaNiO_3−x_ with varying La and Ni compositions was investigated via X-ray diffraction (XRD) analysis using a Rigaku SmartLab diffractometer. X-ray photoelectron spectroscopy (XPS, K-alpha, Thermo, Oxford, UK) and Auger electron spectroscopy (AES, PHI-700, ULVAC-PHI) were conducted to investigate the natures of the chemical bonding and chemical composition in the thin films, respectively. The I–V characteristics of the LaNiO_3−x_ films were measured using DC and AC pulses with a bias of −6.0 to 6.0 V and a time duration of 50–500 ns using a Keysight B1500A analyzer at 25 °C. The compliance current for the DC bias test was set to 100 μA to avoid the hard breakdown of the device. A gradual weight update of a synaptic device was also measured in the consecutive AC pulse mode. For the weight operation of potentiation and depression, voltages of −4.0 and 3.5 V were applied at the same pulse width of 50 ns, respectively.

## 3. Results and Discussion

**Figure 1:**
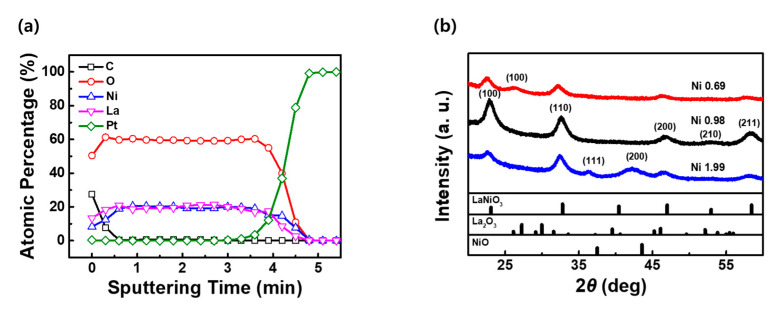
(**a**) Auger electron spectroscopy (AES) depth profile of LaNiO_3−x_ films with a La:Ni ratio of 1:0.98. (**b**) Change in the crystallinity of LaNiO_3−x_ films with various La:Ni ratios.

Figure 1a shows the depth profile of the atomic concentration of the LNO thin film with a La:Ni ratio of 1:0.98, as measured via AES. The chemical composition of the thin film, deposited by co-sputtering using La and Ni targets, was estimated to be La_1_Ni_0.98_O_2.95_, which was similar to that of LNO. Figure 1b shows the XRD patterns of the LNO films with varying La:Ni ratios at an oxygen partial pressure fraction of 25%. Diffraction peaks corresponding to the (100), (110), (200), (210), and (200) crystallographic planes of the LNO structure are observed for the films with a La:Ni ratio of 1:0.98; however, peaks of the La_2_O_3_ and NiO structures are not detected [48,49]. In contrast, LNO and La_2_O_3_ crystalline phases with (100) plane are observed in the diffraction pattern of the films when the La:Ni ratio is 1:0.68, corresponding to Ni-deficient LNO. Similarly, the (111) and (200) diffraction peaks of the NiO phases [50] are observed in addition to the LNO peaks in the XRD pattern of the films when the La:Ni ratio is 1:1.99, corresponding to an LNO film with excess Ni.

**Figure 2:**
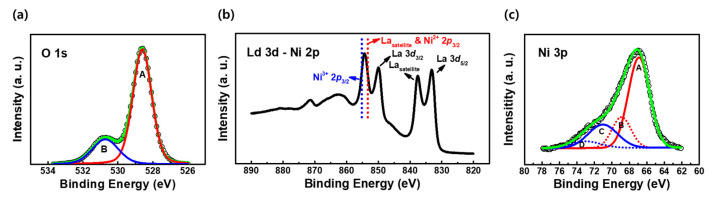
(**a**) Deconvoluted X-ray-photoelectron-spectroscopy (XPS) peak of O 1s in LaNiO_3−x_. The black circle represents the measured peak, whereas the red and blue lines represent the lattice and non-lattice oxygen peaks, respectively. (**b**) XPS profiles of La 3d and Ni 2p. (**c**) Deconvoluted XPS peak of Ni 3p. The black circle represents the measured peak, the solid red peak is the Ni^2+^ 3p_3/2_ main peak, the dashed red peak represents the Ni^2+^ 3p_3/2_ satellite peak, the solid blue line indicates the Ni^3+^ 3p_3/2_ main peak, and the dashed blue line represents the Ni^3+^ 3p_3/2_ satellite peak.

An oxygen deficiency in LNO films leads to an increase in the number of Ni^2+^ ions, which deteriorates the electrical conductivity of LNO [51]. Because the electrical characteristics of the ternary La–Ni–O system depend on the relative ratio between the Ni^3+^ and Ni^2+^ ions in the films, the chemical bonding states of the LNO films were investigated using XPS. Figure 2a shows the deconvoluted O 1s peak of the LNO film with a La:Ni ratio of 1:0.98. The primary peak A, with a lower binding energy of 528.6 eV, is associated with lattice oxygen, and peak B, with a higher energy of 530.7 eV, is associated with oxygen vacancies [52]. The proportion of the areal intensity of peak B was estimated to be 23.2%, which implied a slight oxygen deficiency of the LaNiO_3−x_ thin film. Figure 2b shows the XPS profiles of La 3d and Ni 2p in LaNiO_3−x_ [51,53]. The dotted lines indicate the binding energies of Ni^3+^ 2p_3/2_ and Ni^2+^ 2p_3/2_. Because the XPS peaks of the Ni 2p_3/2_ lines for Ni^2+^ and Ni^3+^ overlap closely with the satellite peak of La 3d_3/2_, the precise estimation of the Ni^3+^/Ni^2+^ ratio is not possible with higher La concentrations in LNO. In contrast, the Ni 3p peak can be easily deconvoluted into Ni^3+^ and Ni^2+^ sub-peaks, although its intensity is relatively lower compared to that of the Ni 2p_3/2_ peak. Figure 2c shows the deconvolution of the Ni 3p peak. The peaks at 66.9 (A) and 71.0 eV (C) are assigned to Ni^2+^ 3p_3/2_ and Ni^3+^ 3p_3/2_, respectively [51,53]. Additionally, peaks B and D are assigned to the satellite components of the Ni^2+^ 3p_3/2_ and Ni^3+^ 3p_3/2_ peaks. The fractions of Ni^3+^ and Ni^2+^ were estimated to be 26.2% and 73.8%, respectively. The chemical composition of the films can be estimated to be oxygen-deficient LaNiO_2.63_, which suggests the existence of a resistive state that can exhibit RS behavior, unlike metallic LNO [51,54].

**Figure 3:**
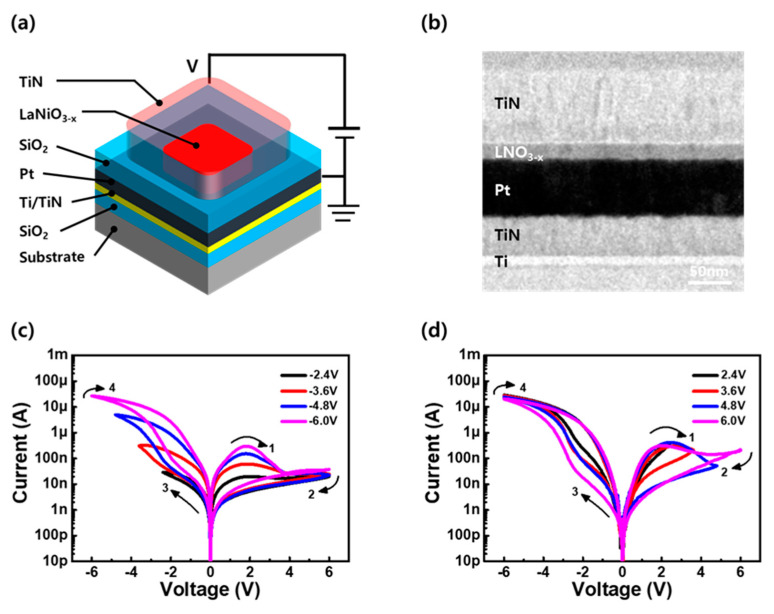
(**a**) Schematic diagram of the LaNiO_3−x_-based device. (**b**) Cross-sectional transmission-electron-microscopy (TEM) image of TiN/LaNiO_3−x_/Pt stacks. Reset-first resistive-switching behavior of LaNiO_3−x_-based devices with modulating (**c**) V_set_ and (**d**) V_reset_.

Figure 3a illustrates the schematic diagram of the LaNiO_3−x_-based device with MIM structure. The structure of the TE TiN/15 nm LaNiO_3−x_/BE Pt stack was observed using cross-sectional transmission electron microscopy (TEM), as shown in Figure 3b. The RS behavior of the TiN/LaNiO_3−x_/Pt device was investigated, and its I–V characteristics in the DC mode are shown in Figure 3c–d. In contrast to conventional RRAM, which requires an electroforming process, the device exhibits reset-first RS behavior under a positive bias. Figure 3c illustrates the change in the I–V curve after modulating the set voltage (V_set_) from −2.4 to −6.0 V for a reset voltage (V_reset_) of 6.0 V. As the applied V_set_ increases from −6.0 to −2.4 V, the ON/OFF ratio of RS after the set operation decreases from 1.4 to 0.1 at a read voltage (V_read_) of 2.0 V. Similarly, Figure 3d shows the I–V characteristics for the V_reset_ modulation from 2.4 to 6.0 V with a V_set_ of −6.0 V. As V_reset_ decreases from 6.0 to 2.4 V, the ON/OFF ratio of RS decreases from 1.5 to 0.2 at a V_read_ of 2.0 V, possibly owing to the insufficient reset of resistance in the high-resistance state (HRS). Compared with the low-resistance state (LRS) in Figure 3c, the current in the HRS owing to the voltage modulation shows an abrupt change as V_reset_ increases, consistent with the typical characteristics of RRAMs in the HRS [17]. Moreover, it shows a typical I–V curve of interface-type RS, similar to that of perovskite-material-based RRAMs, and multibit-level characteristics in which the resistance gradually changes with voltage modulation [55]. The analysis of the LaNiO_3−x_/TiN interface in Figure S2 and the characteristics of area dependence in Figure S3 support the interface-type RS behavior of LaNiO_3−x_-based devices. Based on these results, the presented RS mechanism is shown in Figure S1.

**Figure 4:**
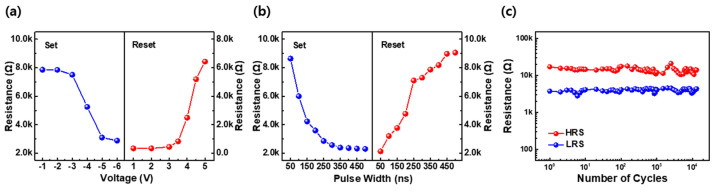
AC pulse characteristics of the TiN/LaNiO_3−x_/Pt device at variable voltages and pulse widths. (**a**) Resistance changes under varying voltage conditions of set and reset pulses with a pulse width of 100 ns. (**b**) Resistance changes under varying pulse-width conditions for a set voltage of −4.0 V and reset voltage of 3.5 V. (**c**) AC endurance of RS at room temperature (25 °C). V_reset_, V_set_, V_read_, and the pulse width were set to 3.5 V, −4.0 V, 0.5 V, and 500 ns, respectively.

The RS characteristics of the LaNiO_3−x_ films were evaluated using variable AC voltages and pulses. Figure 4a shows the resistance changes in the LRS and HRS under pulses of varying voltages and a fixed pulse width of 100 ns. The initial resistance of the HRS remains unchanged for set pulses up to −2 V but starts to decrease when the voltage is above −2 V and eventually saturates for a set voltage above −6 V. The resistance of the LRS increases at a reset-voltage pulse above 3 V. The resistance changes of the reset process due to voltage variations are slightly steeper than those of the set process, consistent with the I–V characteristics obtained in the DC mode. Figure 4b shows the resistance change with a pulse width of 50–500 ns for a reset-voltage pulse of −4.0 V and set-voltage pulse of 3.5 V. The resistance of the LaNiO_3−x_-based devices shows a gradual change with the pulse width for the set and reset operations. The resistance states are completely switched to opposite states for a voltage pulse longer than 400 ns. The ratio of high/low resistances was estimated to be ~4.5 with a resistance difference of approximately 7 kΩ. The AC endurance characteristics of the TiN/LaNiO_3−x_/Pt device are shown in Figure 4c. For the measurement, V_reset_, V_set_, V_read_, and the pulse width were set to 3.5 V, −4.0 V, 0.5 V, and 500 ns, respectively. The AC endurance of RS between the HRS and LRS was maintained at approximately 10^4^. A data retention test was also conducted, as shown in Figure S4. It was maintained during approximately 10^5^ sec without any deterioration and the characteristics of retention estimated by the linear extrapolation was 10 years, indicating high reliability in emerging memory applications.

**Figure 5:**
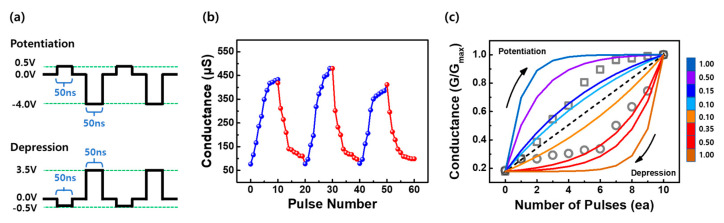
Synaptic potentiation and depression characteristics of the TiN/LaNiO_3−x_/Pt device for consecutive AC pulses. (**a**) Pulse waveforms for the measurement of potentiation and depression. (**b**) Reproducibility of conductance modulation for potentiation (blue) and depression (red) behaviors over three cycles. (**c**) Potentiating (square) and depressing (circle) curves obtained by applying ten consecutive pulses. Analog conductance curves with varying nonlinearity values (*ν*) are presented together, with blue-based colors for potentiation and red-based colors for depression.

Next, we investigated the possibility of neuromorphic computing applications using the LaNiO_3−x_-based device. Figure 5a shows the pulse waveforms used to measure the potentiation and depression of the LaNiO_3−x_ devices for application to synaptic devices. To increase the conductance for potentiation, voltage pulses with an amplitude and width of −4.0 V and 50 ns, respectively, were applied. Voltage pulses with an amplitude of 3.5 V and the same pulse width were used for depression. Figure 5b shows the potentiation and depression behaviors over consecutive multiple cycles. The uniform and reproducible analog switching behavior shows that the conductance of 50~500 μS is maintained for three cycles. Figure 5c illustrates the nonlinear conductance modulation curves of the weight update and calculated various nonlinearity. A progressive change in the conductance of the potentiation is observed with an increasing number of pulses, as demonstrated with the square symbols in Figure 5c. However, the conductance of the depression decays more abruptly with the pulse number, as indicated by the circles in Figure 5c.

Generally, a linear increase/decrease in the conductance with the operating pulses is preferable for neuromorphic devices [56]. However, for most RRAMs, nonlinearity and asymmetry are intrinsic because of their nonuniform filamentary switching behavior. Herein, only the nonlinear characteristics of the LaNiO_3−x_ device were investigated via constant-pulse programming, without applying any techniques to enhance the nonlinear conductance, such as incremental-width-pulse programming and incremental-step-pulse programming [57]. The nonlinearity (*ν*) of the TiN/LaNiO_3−x_/Pt device was estimated using the following equations [58,59]:

(1) $$G_{i}=\frac{G_{max}-G_{min}}{1-e^{{-vP}_{max}}},$$

(2) $$G_{LTP}=G_{min}+G_{i}\left(1-e^{-vP}\right),$$

(3) $$G_{LTD}=G_{max}-G_{i}\left(1-e^{v\left(P-P_{max}\right)}\right),$$

where *G_max_* and *G_min_* are the maximum and minimum conductance values of the LaNiO_3−x_-based device, respectively. *P* and *P_max_* denote the number of input pulses and the maximum pulse number required to switch the device between *G_max_* and *G_min_*, respectively. *G_i_* was used as a fitting parameter. The nonlinearity values of potentiation and depression were estimated to be 0.15 (Figure 5b, blue line) and 0.35 (Figure 5b, red line), respectively. Figure 3a,b show that potentiation has a higher linear tendency than depression, which is consistent with the abrupt resistance change in the reset state. The Δ*G* of the weight levels was estimated to be approximately 50 μS, based on the median value, which was sufficient to distinguish the resistance difference among multi-level states [56]. In general, other studies have reported that nonlinearity values below 0.5 result in an accuracy loss of approximately less than 1% [60]. Therefore, the LaNiO_3−x_-based RRAMs could be used as conventional- and analog-type devices with multiple levels.

## 4. Conclusions

In this study, TiN/LaNiO_3−x_/Pt RRAM devices were fabricated, and their RS characteristics were investigated. The oxygen-deficient LaNiO_3−x_ films, deposited via the RF-magnetron reactive co-sputtering of La and Ni, exhibited conducting properties in the pristine state. The electrical characteristics of these devices showed reset-first RS behavior without an electroforming step, suggesting their potential as low-power devices. Furthermore, the application of an AC pulse led to a gradual increase/decrease in the resistance with the modulation of the voltage and width. The nonlinearity values of potentiation and depression were estimated to be 0.15 and 0.35, respectively, resulting in good performance as synaptic devices. Therefore, the LaNiO_3−x_-based RRAM is one of the potential candidates for the next-generation synaptic device with low power consumption in neuromorphic computing.

## Data Availability

The data reported in this research are available from the corresponding author upon request.

## Supplementary Materials

The following supporting information can be downloaded at: https://www.mdpi.com/article/10.3390/ma16144992/s1, Figure S1: Schematic diagrams of purposed resistive switching mechanism of LaNiO_3−x_-based device; Figure S2: TEM image of TiN/LaNiO_3−x_ interface after TiN deposition, and XPS peaks of Ti 2p analyzed by depth profile technique; Figure S3: Area dependence of LaNiO_3−x_-based devices on LRS and HRS; Figure S4: Retention behavior of RRAM device consisting of LaNiO_3−x_ films at 85 °C.
