# Supplementary material for: Reset-First and Multibit-Level Resistive-Switching Behavior of Lanthanum Nickel Oxide (LaNiO3−x) Thin Films
Source: Materials (Basel). 2023 Jul 14;16(14):4992. doi: 10.3390/ma16144992 (PMC10384036; doi:10.3390/ma16144992)
Supplement: Supplementary file 1 [file materials-16-04992-s001.zip › materials-2493097-supplementary.pdf]

Supplementary materials

Reset-First and Multibit-Level Resistive-Switching Behavior of Lanthanum Nickel Oxide ($LaNiO_{3-x}$) Thin Films

Daewoo Kim, Jeongwoo Lee, Jaeyeon Kim and Hyunchul Sohn *

Department of Materials Science and Engineering, Yonsei University, Seoul 03722, Republic of Korea; daewoo.kim@yonsei.ac.kr (D.K.)
* Correspondence: hyunchul.sohn@yonsei.ac.kr; Tel.: +82-2-2123-5850

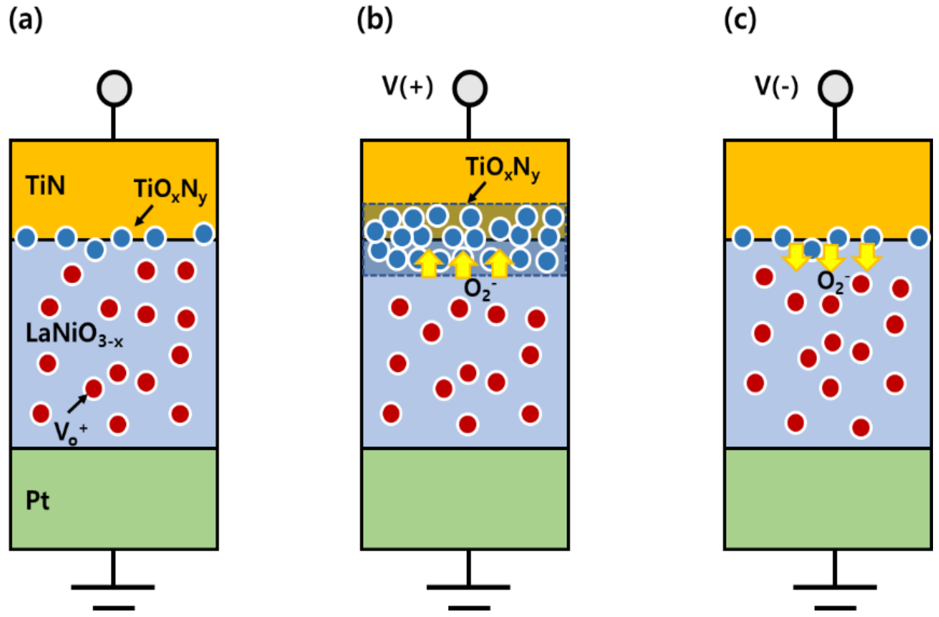

Figure S1. Schematic diagrams of purposed resistive switching mechanism of $LaNiO_{3-x}$-based device (a) Pristine state with randomly distributed oxygen vacancies in $LaNiO_{3-x}$ with TiO_xN_y at TiN/$LaNiO_{3-x}$ interface during the deposition (b) Reset operation with enhanced TiO_xN_y barrier by O_2^- moving to the top electrode. (c) Set operation by O_2^- moving toward the $LaNiO_{3-x}$ thin film.

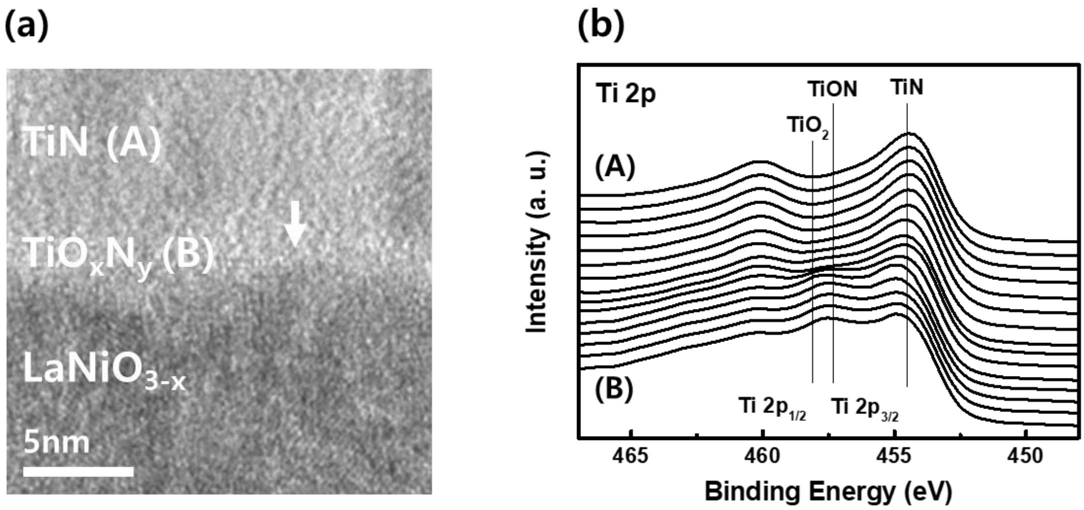

Figure S2. (a) TEM image of TiN/$LaNiO_{3-x}$ interface after TiN deposition, and (b) XPS peaks of Ti 2p analyzed by depth profile technique.

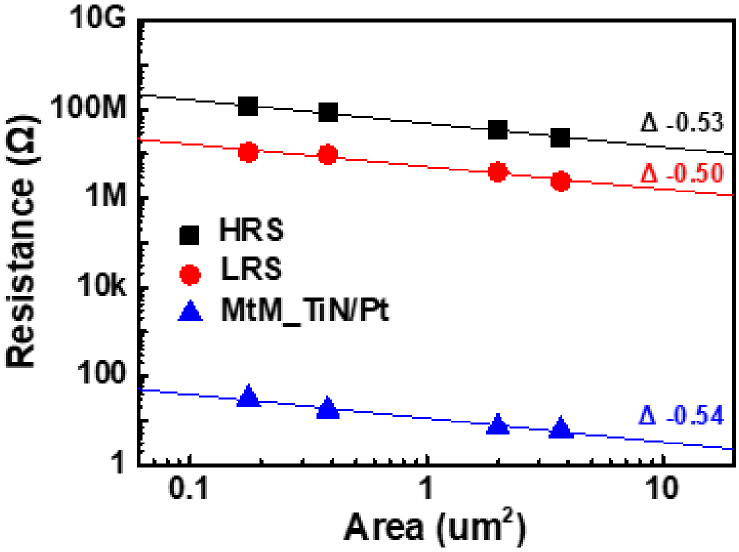

Figure S3. Area dependence of $LaNiO_{3-x}$-based devices on LRS and HRS. The metal-metal junction without $LaNiO_{3-x}$ films is also presented as reference. The slope (Δ) value was estimated using the linear fitting method.

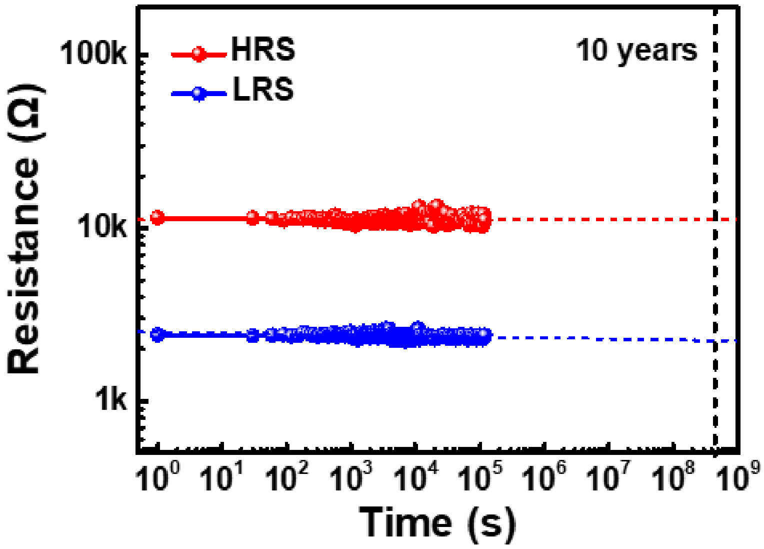

Figure S4 Retention behavior of RRAM device consisting of $LaNiO_{3-x}$ films at 85℃. Set voltage (V_{set}), reset voltage (V_{reset}), read voltage (V_{read}) and pulse width were set to -4.0 V, 3.5 V, 0.5 V, and 500 ns respectively.
